# Exploration on the bio-recycling agent and its recycled asphalt: Composition ratio, recycling efficiency and micro-mechanism

**DOI:** 10.1371/journal.pone.0334052

**Published:** 2025-10-09

**Authors:** Ying Fang, Zhengqi Zhang, Fei Zhou, Hengbin Liu, Zhongnan Tian

**Affiliations:** 1 School of Civil Engineering and Architecture, Hubei University of Arts and Science, Xiangyang, Hubei, China; 2 Hubei Key Laboratory of Vehicle-infrastructure Cooperation and Traffic Control, Hubei University of Arts and Science, Xiangyang, Hubei, China; 3 Key Laboratory for Special Area Highway Engineering of Ministry of Education, Chang’an University, Xi’an, Shaanxi, China; 4 School of Transportation and Civil Engineering, Shandong Jiaotong University, Ji’nan, Shandong, China; 5 Hebei Provincial Communications Planning, Design and Research Institute Co., Ltd., Shijiazhuang, Hebei, China; Shandong University of Technology, CHINA

## Abstract

Mineral oil-based asphalt recycling agents pose environmental concerns, prompting the search for sustainable alternatives. To this end, a bio-recycling agent (BRA) based on waste vegetable oil (WVO) was initially designed for the utilization of recycled asphalt pavement (RAP) through the orthogonal test, and then its physical-chemical and permeating properties were analyzed by a series of tests. Next, the bio-recycled asphalt binder was prepared and its several pavement behaviors were measured via adhesion, rheology and fatigue experiments, thus its recycling efficiency was validated. Ultimately, the microscopic mechanism of bio-recycled asphalt was revealed with differential scanning calorimetry (DSC) and atomic force microscope (AFM) tests. Research results showed that the composition of BRA was determined as base oil components: permeation components: polymer components: functional components = 120: 2: 13: 2.4. Compared to two commercial recycling agents (LBS and HRA), BRA possessed better recycling effect at the same dosage, which obviously restored the adhesion, high- and low-temperature rheological properties and fatigue resistance of aged asphalt, and the BRA increased fatigue life of aged matrix asphalt binder by about 60% compared to LBS at 9% dosage. Moreover, the recycled asphalt with suitable BRA dosage can adapt to a wider range of traffic grades than original asphalt, and for aged matrix and SBS-modified asphalt binder, it was recommended that the reasonable dosage of BRA was 7%−9% and 3%−5%, respectively. The samples recycled by three recycling agents all exhibited favorable thermal stability, and which was basically the same as or higher than that of the original asphalt. Besides, after incorporating the BRA, the number and size of bee-structures in aged asphalt decreased, while the roughness of asphalt surface slightly descended. Meanwhile, compared to two-commercial recycling agents, BRA had a significant improvement on the micro adhesion of aged asphalt, and behaved a better recycling effect on the aged asphalt.

## 1 Introduction

With the extension of road service duration, asphalt pavement built previously will continue to enter the stage of maintenance and expanded-construction. In this process, it is inevitable to produce the waste asphalt mixtures, and their stacking will cause the occupation of space and be disadvantageous to environmental protection. Thus, how to deal with them scientifically and efficiently has become the focus of scholars in road field in recent decades. Fortunately, recycling these waste asphalt mixtures followed by repaving asphalt pavement under the high-temperature construction conditions is one of the effective means to achieve their disposal. To realize the favorable regeneration, a recycling agent is a crucial component in the recycling of waste asphalt mixtures, and its recycling efficiency significantly affects the pavement performance and long-term durability of recycled asphalt mixtures [1,2]. Currently, mineral oil-based recycling agents are chiefly applied to recycle the waste asphalt mixtures, whose main constituent, i.e., base oil, is refined from non-renewable petroleum products, thus it is environmentally unfriendly and has been found to be uneven recycling performance [3–5], imposing certain restrictions on its application with the continuous deepening and implementation of green roads and sustainable development construction concepts. Under this background, seeking environmentally-friendly and efficient recycling agents has become a direction that road practitioners continue to try and practice.

In recent years, the emergence of bio-recycling agents has opened up fresh ideas for the environmentally-friendly and efficient recycling of waste asphalt materials. Bio-recycling agents are developed based on bio-oil (including plant straw, animal and plant waste, microorganisms, waste vegetable oil, and gutter oil, etc), showing favorable environmental protection effect. Among which, bio-recycling agents with waste vegetable oil (WVO) as the key component are a major type of biological rejuvenators, and have attracted the close attention from many researchers [6,7]. At present, to promote the industrial application of WVO bio-recycling agents, their feasibility for the recycling of waste asphalt materials has been first analyzed and confirmed. For instance, Elkashef et al. [8] and Podolsky et al. [9] added soybean oil based-recycling agents to different polymer-modified asphalts, confirming the application prospect of this soybean oil material as a recycling agent. Similarly, Zahoor et al. [10] pointed out that WVO, as an environmentally-friendly material, can be used for the recycling of waste asphalt materials, and it had a promising application prospect for thermal recycling of asphalt pavements.

Based on this, the recycling effect of WVO-based rejuvenators has also been paid attention to scholars. For example, Su et al. [11] studied the feasibility of using waste vegetable oil as a recycling agent for aged asphalt and concluded that WVO recycling agents can restore a significant degree of the pavement properties of aged asphalt. Blanc et al. [6] found that the improvement effect of WVO recycling agents on the performance of waste asphalt mixtures was better than that of mineral oil recycling agents. Ruan et al. [12] pointed that WVO was compatible with aged asphalt binder molecules and can improve their physical and high-/low-temperature properties, and similarly, Zhang et al. [13] and Bastola and Teixeira [14] indicated that the vegetable oils in waste cooking oil enhanced low-temperature flexibility and fatigue resistance of aged asphalt binder. Further, Zhao et al. [15] found that the WVO with strong fluidity and weak volatility exhibited favorable rejuvenation effects and aging resistance, providing a scientific screening scheme for WVO. Forton et al. [16] analyzed the influence of a bio-rejuvenator on the mechanical properties of asphalt mixtures, and announced the relationship between the mechanical behaviors of asphalt mixtures and the penetration values of corresponding binders. Besides, Beghetto [17] and Zhang et al. [18] reviewed the several pavement properties of recycled asphalt binders using bio-rejuvenators as a green roadway material, and confirmed the positive effects of WVO in restoring the properties of aged asphalt. However, according the previous documents, despite the beneficial effects of WVO agent, it was found that the recycling effect of WVO-based recycling agents on aged asphalt still needed to be improved for future large-scale practical application, especially when agent was used alone for the recycling of aged asphalt without adding other additives [19,20]. In view of this, in order to balance environmental friendliness and satisfactory recycling effect, it is necessary to develop high-performance WVO-based recycling agent and explore its usage behaviors.

For developing the high-performance WVO-based recycling agent, according to the theory of composite materials science, some additives should be added into the base oil (WVO) to achieve synergistic effects and realize good recycling effect. Nevertheless, there are currently few studies exploring the optimized composition design of WVO rejuvenator after adding additives; further, the bio-rejuvenator with added additives will exhibit different rejuvenation effects, and it is necessary to investigate its rejuvenation effect and microscopic mechanism to theoretically guide its promotion and application.

Under the background mentioned above, the research aims to explore the optimized composition of WVO rejuvenator with incorporating additives, and further to analyze its recycling efficiency and microscopic mechanism. For fulfilling these purposes, initially, the base oil components, permeation components, polymer components and functional components were selected as raw materials for the composition of recycling agents in the research, the composition ratio of bio-recycling agent (BRA) was determined through orthogonal experiments, and the generally characteristics of BRA were evaluated. On this basis, the influence of different BRA dosages on the performance of aged asphalt was explored and its recycling efficiency was assessed. Finally, the microscopic recycling mechanism was revealed through a series of micro testing techniques. The research results contribute to promoting the high-value recycling of waste asphalt materials and have remarkable economic and environmental benefits.

## 2 Materials and methods

### 2.1 Test materials

#### 2.1.1 Aged asphalt.

The SK-70# and SBS-modified asphalt binders were selected as original asphalts in the study. The original asphalts were subjected to an aging process of RTFOT (Rolling Thin Film Oven Test) with a duration of 85 min, followed by a PAV (Pressure Aging Vessels) test for 20h to prepare aged asphalt samples for subsequent experiments. Table 1 shows the technical indicators of two asphalt binders.

**Table 1:** Basic technical indicators of asphalt binders.

Technical indicators	Unit	SK-70# asphalt		SBS modified asphalt		Test methods
		Original	Aged	Original	Aged	
Penetration (25°C)	0.1 mm	70.5	23.3	55.5	27.7	T-0604
Softening point	°C	46.8	61.2	75.5	86.9	T-0606
Ductility	cm	50.8	1.6	25.5	0.9	T-0605
Viscosity (135°C)	Pa·s	0.455	1.42	1.970	2.856	T-0625

The temperatures of ductility test for matrix asphalt and modified asphalt are 10°C and 5°C, respectively.

#### 2.1.2 Recycling agent.

Two commercial recycling agents, named LBS and HRA, were selected as the control group recycling agents, with their technical indexes shown in Table 2.

**Table 2:** Technical indicators of two commercial recycling agents.

Test items	LBS	HRA	Technical requirement
Viscosity/Pa·s (25°C)	1.206	1.650	0.1 ~ 20 Pa·s
Surface tension/mN·m^-1^ (25°C)	34.28	33.39	mN/m
Aromatics/%	81.7	35.2	≥30
TFOT viscosity ratio	1.34	2.42	<3
TFOT mass variation/%	0.5	0.8	—

#### 2.1.3 Constituent materials of bio-recycling agents.

(1) Base oil

Base oil in the research is a mixture of kitchen waste oil and plasticizer, with a mass ratio of 100: (15 ~ 25). Kitchen waste oil belongs to aromatic oils, with small molecular weight, high surface tension, low viscosity and high aromatic content, which can fully and quickly blend with aged asphalt and exert its recycling function. The plasticizer selected is acetyl tributyl citrate (ATBC), which is a commonly used plasticizer for asphalt binder and its derivatives. Due to the uncertainty and the uncontrollable nature of the collected waste oil, the laboratory simulated cooking aged soybean oil was selected as the base oil in this research, which was obtained by continuously heating fresh soybean oil in 180°C oven for 6 hours. Table 3 lists the main technical indicators of the simulated-aged soybean oils.

**Table 3:** Basic technical indicators of waste soybean oil.

Appearance	Moisture content/%	25°C density/g·cm^-3^	60°C viscosity/Pa·s
Yellow liquid	0.141	0.897	0.0571

(2) Permeation component

Permeation component is worked to promote the rapid and sufficient fusion between recycling agent and aged asphalt, and non-ionic surface active substances are amphiphilic (hydrophilic and oleophilic) compounds with excellent wetting and permeation functions, making them an alternative permeation component. In this research, the non-ionic surface active substance alkylphenol polyoxyethylene ether (OP-10) (APEO), which is considered to be excellent dispersibility, wettability and permeability, was selected as the permeation component of recycling agent to improve the permeability of the base oil, and its main technical indicators are shown in Table 4.

**Table 4:** Technical indicators of alkylphenol polyoxyethylene ether (OP-10).

Appearance	Active content	Moisture content	Cloud point	PH-value	HLB	CMC/ Surface tension
Transparent liquid	99.90%	0.06%	65 ± 3°C	6.0-7.5	13.2	45/28

(3) Polymer component

Polymer component is incorporated to adjust the viscosity and high-temperature stability of the recycling agent and recycled asphalt. Research has shown that tackifying resin can effectively reduce the intermolecular forces between asphaltenes in aged asphalt binder, thus improving their mutual motion and fluidity, which is crucial for achieving the rejuvenation of aged asphalt binder. For conducting the research, the commonly used tackifying resin named FTR-6100 aromatic petroleum resin, which has the advantage of favorable adaptability to organic asphalt materials, was selected to enhance the recycling effect of the light oil recycling agent, and its specific technical indicators are shown in Table 5.

**Table 5:** Technical indicators of tackifying resin (FTR-6100).

Appearance	Softening point	Viscosity	Density	Molecular weight Mw/Mn
White block particles	95°C	60 mPa ∙ s	1.03 g/cm^3^	1.40

(4) Functional component

For improving the secondary anti-aging ability of recycled asphalt and ensure a satisfactory miscibility of each component with the base oil, the functional component needs to be added during the development of recycling agent. In the study, the functional component is a compound of BASF antioxidant 1010 and KH-550 silane coupling agent, with a mass ratio of 1: 3. Among which, BASF 1010 and KH-550 are typical anti-aging agent and compatibilizer in materials science, respectively, and they can be stably blended with asphalt components. Thus, they were screened as the functional component for research.

### 2.2 Test methods

#### 2.2.1 Preparation of bio-recycling agent and recycled asphalt.

(1) Preparation of bio-recycling agent (BRA)

The waste soybean oil and plasticizer with a mass ratio of 100: (15 ~ 25) were poured into a container, heating to 135°C and stirring evenly with a glass rod to obtain the base oil component. Then, maintaining the temperature and adding the permeation component APEO, polymer component FTR-6100 and functional component in sequence. Subsequently, stirring the blend with a stirrer for 30 minutes to be evenly mixed, and thus the bio-recycling agent can be obtained after the blend cooled to room temperature.

(2) Preparation of recycled asphalt

**Fig 1:**
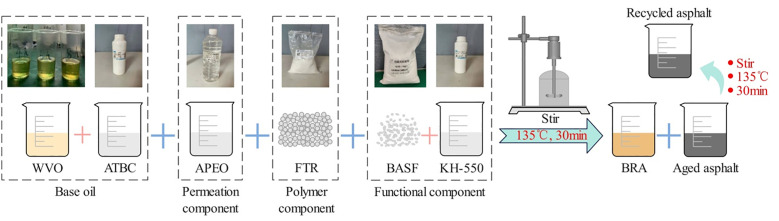
Schematic for preparation steps of BRA and recycled asphalt.

A certain amount of aged asphalt was poured into a container and heated to a molten state at 135°C, and meanwhile, the recycling agent was heated and controlled to about 135°C. Then, maintaining the temperature at 135°C, 7% of the recycling agent was incorporated to the aged asphalt, and the blend was stirred at 300 prm continuously for about 30 minutes by using a spiral mixer until the recycling agent was uniformly mixed with the asphalt. Eventually, the recycled asphalt can be obtained after the mixture cooled to room temperature. Fig 1 shows the schematic for preparation steps of BRA and recycled asphalt.

#### 2.2.2 Orthogonal design test.

Orthogonal design test was executed to analyze the influence of constituent materials of bio-recycling agent on the pavement properties of recycled asphalt, thus determining the optimized proportion of constituent materials of bio-recycling agent. When implementing an orthogonal design test, the number of influencing factors and levels of each influence factor needs to be considered in advance. Because the WVO-based bio-recycling agent in this study includes the four components, namely base oil, permeation component, polymer component and functional component, there are four factors affecting the rejuvenation effects of the WVO-based bio-recycling agent. Further, according to the theory of orthogonal design test, the number of levels of influencing factors determines the resultant accuracy and experimental group size. A smaller number of factor levels means smaller experimental groups, which can simplify the experiment process, but at the same time, the resultant accuracy may not be ideal; conversely, a larger number of factor levels can effectively improve the accuracy of the results, while increasing the trial groups. For this reason, an orthogonal experiment with four factors and four levels (L_16_ (4^4^)) was ultimately selected for this research through balancing the contradiction between resultant accuracy and experimental group size. Besides, the level range of influencing factors was determined based on preliminary experiments and similar research experience to carry out this study.

#### 2.2.3 Dynamic shear rheological test.

The high-temperature shear rheological test of recycled asphalt was conducted using the SmartPave102 dynamic shear rheometer (DSR) produced by Anton Paar, Austria. Multiple stress creep recovery (MSCR) test and Linear amplitude sweeping (LAS) test were conducted on the recycled asphalt samples to evaluate their high-temperature rheological properties and fatigue resistance. Among them, in the MSCR test, a 25 mm rotor model was selected, with a gap set to 1 mm, and the tests at the temperatures of 58°C, 64°C and 70°C were performed — as for one temperature, two stress levels of 0.1kPa and 3.2kPa were configured for trialing, respectively. In the LAS test, an 8 mm parallel plate was applied, and the entire testing process was divided into two stages: (1) the first stage was the frequency sweeping stage in the range of 0.1–30 Hz; and (2) the second stage was the amplitude sweeping stage. Before conducting the experiment, the asphalt binder was initially subjected to RTFOT and PAV tests to simulate the aging process of asphalt during mixing, paving and usage, with a setting temperature of 25°C. All the above tests were conducted in accordance with the ASTM D7405-24 and AASHTO T391-20 specifications. As for each test, three replicates were conducted to minimize the variability of the experimental results, and the results were the mean of the three repeated trials.

#### 2.2.4 Differential scanning calorimetry test (DSC).

**Fig 2:**
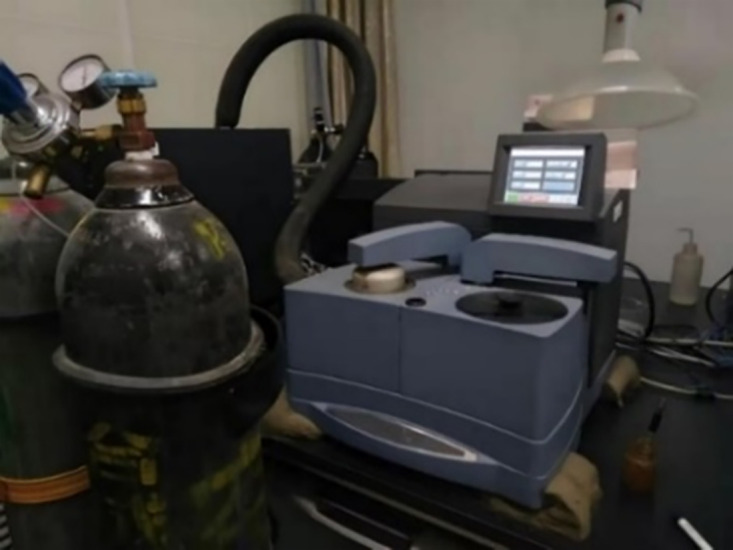
Differential scanning calorimetry tester.

Differential Scanning Calorimetry (DSC) is a technique used to measure the energy difference and temperature relationship between a sample and a reference material under programmed temperature control. On the DSC curve, the difference in absorbed heat is reflected as the area contained in the curve (known as enthalpy change △H). Generally, the larger the enthalpy change △H in a certain temperature range, the more unstable the material is in a multiphase mixing state. On the DSC curve, the enthalpy change size and endothermic peak position can be extracted, which are two important indicators for evaluating the thermal properties of asphalt binder. The DSC testing instrument is shown in Fig 2. Besides, it was worth noting that when conducting the DSC test, three repeated tests were executed on a sample to reduce the variability of experimental data, and the average of the three repeated test results was used as the final outcome for analysis.

#### 2.2.5 Atomic force microscope (AFM) test.

**Fig 3:**
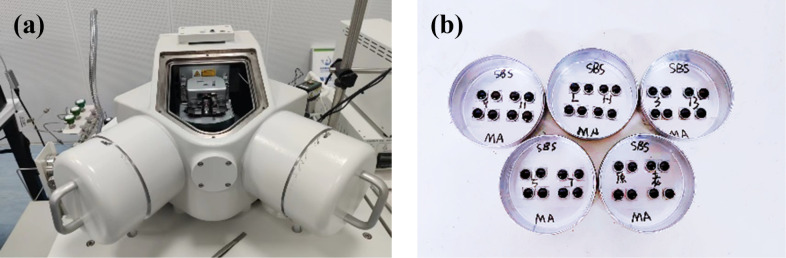
AFM test equipment and samples. (a) Test equipment; (b) Test samples.

The AFM test equipment used in this study is the SPM-9700 atomic force microscope from Shimadzu Corporation in Japan. The main test parameters are: a) scanning range of 20 μm × 20 μm, b) scanning frequency of 2 Hz, c) resolution of 256 × 256, d) NCHR-10 probe with a thickness of 4 μm, length of 125 μm, width of 30 μm, and e) nominal elastic coefficient of 42 N/m. Considering the characteristics of each scanning mode and the limitations of practical conditions, the tapping mode was used to test the nano morphology characteristics of the samples, and the contact mode was applied to test the nano force curve of the samples to analyze their phase mechanical properties, with the assumption that while the probe came into contact with the sample surface, the probe followed the law of elastic deformation; meanwhile, when obtaining the force curve, the elastic coefficient of the probe was first calibrated using a sapphire through the balance method, and the actual elastic constant was used as the input parameter for measuring mechanical index. Besides, as for each sample, it was subjected to three repeated tests to weaken the influence of data variability, and for adhesion force data, their average was obtained as the final output for analysis. Fig 3 displays the AFM test equipment and samples used.

## 3 Results and discussions

### 3.1 Composition ratio analysis of recycling agent based on orthogonal test

The orthogonal test table L_16_ (4^4^) for the composition materials of recycling agent is shown in Table 6 according to orthogonal design test. Based on the dosage of each material, the bio-recycled asphalt (the dosage of recycling agent was 8%) was prepared and its basic properties were measured and documented in Table 7.

**Table 6:** The orthogonal test table L_16_ (4^4^).

Factor level	Base oil/ g	Permeation components/ g	Polymer components/ g	Functional components/ g
1	100	2	5	0.8
2	110	3	9	1.2
3	120	4	13	1.6
4	130	5	17	2.4

**Table 7:** The orthogonal test results.

No.	Base oil/ g	Permeation components/ g	Polymer components/ g	Functional components/ g	Penetration/0.1 mm	Softening point/°C	Ductility (cm, 10°C)	Viscosity (Pa·s, 135°C)
1#	100	2	5	0.8	73.3	47.3	34.2	0.567
2#	100	3	9	1.2	65.1	52.3	35.4	0.445
3#	100	4	13	1.6	70.3	48.2	30.1	0.610
4#	100	5	17	2.4	71.5	49.5	27.2	0.695
5#	110	2	9	1.6	66.1	50.1	48.6	0.482
6#	110	3	5	2.4	75.1	44.3	43.2	0.523
7#	110	4	17	0.8	74.3	45.7	41.1	0.637
8#	110	5	13	1.2	77.4	47.5	37.5	0.582
9#	120	2	13	2.4	68.4	51.6	58.1	0.456
10#	120	3	17	1.6	71.2	50.4	50.3	0.525
11#	120	4	5	1.2	79.8	43.2	55.6	0.467
12#	120	5	9	0.8	78.3	45.7	52.5	0.511
13#	130	2	17	1.2	75.4	49.5	43.2	0.685
14#	130	3	13	0.8	70.2	47.3	47.5	0.581
15#	130	4	9	2.4	80.1	44.1	40.1	0.532
16#	130	5	5	1.6	78.2	41.2	38.2	0.522

**Fig 4:**
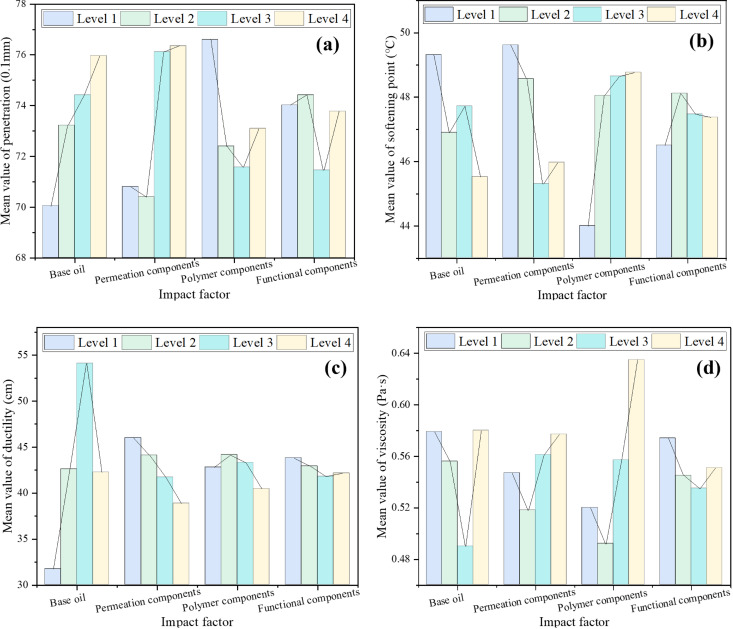
The mean histogram of each index of recycled asphalt. (a) Mean value of penetration; (b) Mean value of softening point; (c) Mean value of ductility; (d) Mean value of viscosity.

The mean values of the test results of each factor at each level were calculated respectively, and the mean histograms of penetration, softening point, ductility and viscosity of each factor at different levels were drawn respectively, as shown in Fig 4, to analyze the influence of each factor on the three major indicators and viscosity.

According to the principle that the index values of recycled asphalt are close to those of original asphalt, the composition ratio of BRA was determined using intuitive analysis method. According to Fig 4(a), the range order of penetration mean-values for different levels of each factor was as follows: R _base oil_ > R _permeation components_>R _polymer components_>R _functional components_, indicating that the impact of these four factors on penetration was sorted by base oil, permeation components, polymer components and functional components in descending order. Based on the principle of low penetration of recycled asphalt, it was recommended that the optimal composition ratio of the recycling agent be 2 #, 5 #, and 9 #. From Fig 4(b), it can be seen that the range order of softening points mean-values for different levels of each factor was R _polymer components_ >R _permeation components_>R _base oil_ > R _functional components_, demonstrating that the order of the impact of these four factors on softening points was: polymer components>permeation components>base oil>functional components. According to the principle of high softening point of recycled asphalt, the recommended optimal composition ratios for recycling agents were 2 #, 5 #, 9 #, and 10 #. According to Fig 4(c), the range order of the ductility mean-values for different levels of each factor was R _base oil_  > R _permeation components_>R _polymer components_>R _functional components_, signifying that the base oil had the greatest influence on the ductility, followed by permeation components, polymer components and functional components. Based on the principle of high ductility of recycled asphalt, the recommended optimal composition ratios of recycling agents were 9 #, 10 #, 11 #, and 12 #. According to Fig 4(d), the range of viscosity mean-values for different levels of each factor was ranked as follows: R _polymer components_>R _base oil_ > R _permeation components_>R _functional components_, illustrating that the polymer components affected the viscosity most, followed by base oil, permeation components and functional components. Further, it was found that the viscosity of recycled asphalt reached the ideal viscous degree of original matrix asphalt binder (about 0.3 ~ 0.7 Pa·s), and according to the principle that the viscosity of recycled asphalt was approached to that of original neat asphalt, the recommended optimal composition ratios for recycling agents can be determined as 2 #, 5 #, 9 #, and 11 #. In summary, 2#, 5#, 9#, 10#, 11# and 12# schemes were screened for further analysis, whose results were extracted as listed in Table 8.

**Table 8:** Test results of 2#, 5#, 9#, 10#, 11# and 12# schemes.

Schemes	Base oil/ g	Permeation components/ g	Polymer components/ g	Functional components/ g	Penetration/0.1 mm	Softening point/°C	Ductility (cm, 10°C)	Viscosity (Pa·s, 135°C)
2#	100	3	9	1.2	65.1	52.3	35.4	0.445
5#	110	2	9	1.6	66.1	50.1	48.6	0.482
9#	120	2	13	2.4	68.4	51.6	58.1	0.456
10#	120	3	17	1.6	71.2	50.4	50.3	0.525
11#	120	4	5	1.2	79.8	43.2	55.6	0.467
12#	120	5	9	0.8	78.3	45.7	52.5	0.511

For obtaining the optimal scheme, the Technique for Order of Preference by Similarity to Ideal Solution (TOPSIS) was utilized in this study, and its specific calculation procedures, which chiefly included three steps of indicator normalization followed by standardization and score calculation, are presented in [21,22]. When executing the calculation, the penetration was a cost index, the softening point and ductility were benefit indicators and the viscosity was an intermediate target, with the weight of 0.25 of each indicator. Thus, based on the TOPSIS method, the normalized scores for 2#, 5#, 9#, 10#, 11# and 12# were calculated to be 0.6290, 0.7075, 0.8842, 0.5002, 0.4598 and 0.3622, respectively, which manifested that 9# scheme had the highest score, that is, 9# scheme, i.e., 120g of base oil, 2g of permeation components, 13g of polymer components, and 2.4g of functional components, was selected as the final composition ratio of the bio-recycling agent.

### 3.2 Analysis of the basic characteristics of bio-recycling agent

#### 3.2.1 Analysis of physical-chemical properties.

**Fig 5:**
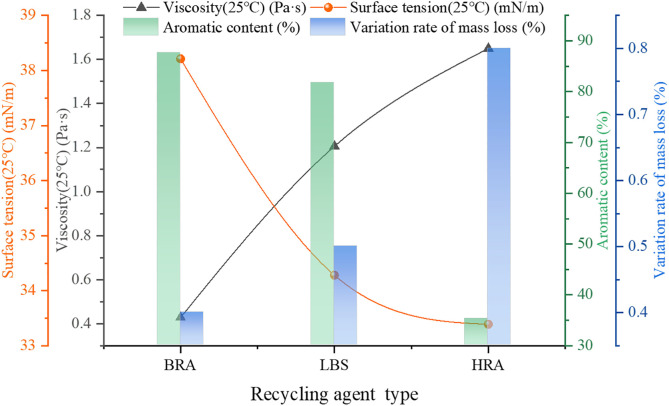
Physical-chemical properties of recycling agents.

In order to investigate whether the conventional technical properties of the developed BRA meets the requirements, two commercially available recycling agents (LBS, HRA) were selected as comparison to explore and evaluate the viscosity, surface tension, aromatic content, ageing resistance and other physical-chemical properties of BRA. The specific test results are shown in Fig 5.

From Fig 5, it can be seen that the viscosity (0.1 ~ 20 Pa·s) and surface tension (≥36 mN/m) of the three recycling agents all met the requirements. Compared to the two-commercial recycling agents, BRA displayed lower viscosity, indicating more favorable fluidity. This was mainly because BRA referred to a light component recycling agent, and its base oil belonged to waste soybean oil that possessed low viscosity and good fluidity, which was conducive to improve the low-temperature crack resistance of aged asphalt. Further, BRA had higher surface tension, which commonly posed BRA a smaller contact angle and greater wettability with asphalt binder, thus the higher surface tension is beneficial for the permeation behavior of BRA and aged asphalt and is essential for achieving the regeneration of aged asphalt binder. In addition, the order of aromatic content of three recycling agents from high to low was: BRA > LBS > HRA, demonstrating that BRA had the highest aromatic content and met the technical requirements (≥30%). According to the asphalt aging principle and component adjustment theory, the aging of asphalt binder was accompanied by the loss of lightweight components (such as aromatics) and the BRA with rich aromatic content can effectively compensate for the missing aromatic components in aged asphalt, restore the component distribution in asphalt, thus having a good recycling effect. Meanwhile, the mass loss rate of BRA was lower than that of the two-commercial recycling agents, implying that the aging resistance and stability of BRA were optimal, and the usage of BRA to rejuvenate aged asphalt was effective in upgrading the anti-aging performance and stability of aged asphalt.

#### 3.2.2 Analysis of permeation characteristics.

**Fig 6:**
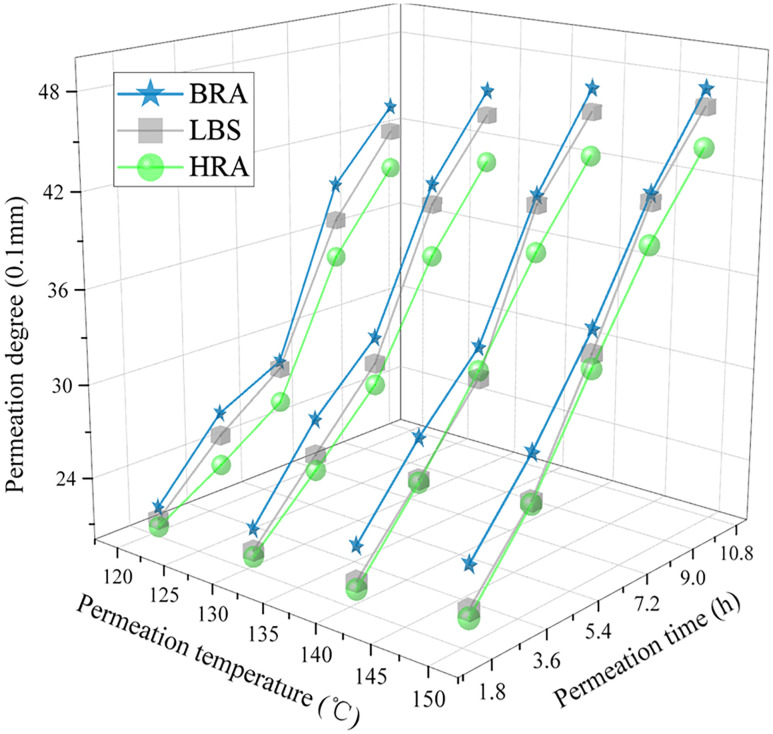
Permeation performance test results of BRA.

In this research, the penetration variation of asphalt (permeation degree) was used to indirectly evaluate the permeability of recycling agent in aged asphalt during the permeation process. According to the method described in reference [23], the experiments were conducted at the temperatures of 120°C, 130°C, 140°C, and 150°C, with permeation times of 2h, 4h, 6h, 8h, and 10h, respectively. The test results are shown in Fig 6.

As shown in Fig 6, the permeation degree of three recycling agents in aged asphalt increased with the increase of permeation temperature or permeation time, indicating that permeation temperature and permeation time possessed a remarkable impact on the permeability of recycling agents in aged asphalt. The higher permeation temperature or the longer permeation time contributed to an excellent permeation effect and an adequate fusion between recycling agent and aged asphalt. In addition, under the same permeation time and temperature conditions, the permeation degree of BRA in aged asphalt was higher than that of the recycling agents (LBS and HRA), explicating that BRA possessed favorable permeation effect in aged asphalt and demonstrated the optimum permeation performance. Considering the actual engineering situation, under the high temperature conditions of mixture mixing and transportation, BRA can fully integrate with aged asphalt in a short period of time due to its prime permeation effect. Meanwhile, in the later paving, rolling and using process, BRA can still effectively penetrate into the aged asphalt layer for a long time and fully integrate with it under the condition of lower temperature, thus achieving favorable recycling effect.

### 3.3 Analysis of the recycling efficiency of bio-recycling agent on aged asphalt

#### 3.3.1 Adhesion analysis.

Adhesion is a key indicator for evaluating the recycling effect of aged asphalt, and as for this study, it is crucial to explore the influence of BRA on the adhesion of aged asphalt. In the current standard, i.e., Standard Test Methods of Bitumen and Bituminous Mixtures for Highway Engineering of China numbered as JTG E20-2011, the water boiling method is used to evaluate the adhesion of asphalt and aggregate. The evaluation indexes of this method are relatively rough, and its 5-level grading index barely distinguish asphalt with similar adhesion levels. In order to make up for this deficiency, researchers have proposed an improved water boiling method and adhesion grading standard by extending the boiling time [24], as shown in Table 9. In view of this, an improved boiling method was applied in this study to investigate the adhesion variation of aged asphalt with the addition of BRA. The specific test results are shown in Table 10.

**Table 9:** Grading standards for improved water boiling method.

Spalling area (%)	Adhesion level	Spalling of asphalt on aggregate surface
≤5	Ⅹ	Asphalt film is intact.
5 ~ 10	Ⅸ	Asphalt film is intact, and there is minimal spalling on the aggregate surface. aggregate surface aggregate surface.
10 ~ 15	Ⅷ	Asphalt film is basically intact, and some aggregates have a small amount of spalling. spalling spalling.
15 ~ 20	Ⅶ	Asphalt film is partially spalling, and the spalling area is between 15% and 30%.
20 ~ 30	Ⅵ	
30 ~ 40	Ⅴ	A large number of asphalt film is spalling, and the spalling area ranges from 30% ~ 60%.
40 ~ 50	Ⅳ	
50 ~ 60	Ⅲ	
60 ~ 70	Ⅱ	Asphalt film is spalling in large area, and the spalling area exceeds 60%.
>70	Ⅰ	A large area of asphalt film is spalling, and the spalling area is more than 70%.

**Table 10:** Adhesion test results of different recycled asphalt samples.

Recycled asphalt	Adhesion level											
	Limestone				Basalt				Granite			
	6 min		15 min		6 min		15 min		6 min		15 min	
	SK	SBS	SK	SBS	SK	SBS	SK	SBS	SK	SBS	SK	SBS
OA	Ⅸ	Ⅹ	Ⅷ	Ⅸ	Ⅷ	Ⅹ	Ⅵ	Ⅸ	Ⅴ	Ⅶ	Ⅲ	Ⅴ
AA	Ⅴ	Ⅶ	Ⅳ	Ⅵ	Ⅳ	Ⅵ	Ⅲ	Ⅵ	Ⅱ	Ⅲ	Ⅰ	Ⅱ
3%-BRA	Ⅶ	Ⅷ	Ⅴ	Ⅶ	Ⅵ	Ⅶ	Ⅳ	Ⅶ	Ⅲ	Ⅵ	Ⅱ	Ⅱ
5%-BRA	Ⅶ	Ⅷ	Ⅵ	Ⅶ	Ⅵ	Ⅶ	Ⅳ	Ⅶ	Ⅲ	Ⅵ	Ⅱ	Ⅲ
7%-BRA	Ⅷ	Ⅸ	Ⅵ	Ⅶ	Ⅵ	Ⅷ	Ⅴ	Ⅶ	Ⅲ	Ⅴ	Ⅱ	Ⅲ
9%-BRA	Ⅷ	Ⅸ	Ⅶ	Ⅷ	Ⅶ	Ⅷ	Ⅴ	Ⅶ	Ⅳ	Ⅴ	Ⅲ	Ⅳ
11%-BRA	Ⅷ	Ⅷ	Ⅶ	Ⅶ	Ⅶ	Ⅶ	Ⅵ	Ⅵ	Ⅳ	Ⅴ	Ⅲ	Ⅳ
13%-BRA	Ⅶ	Ⅶ	Ⅴ	Ⅵ	Ⅴ	Ⅵ	Ⅴ	Ⅵ	Ⅳ	Ⅳ	Ⅱ	Ⅲ
LBS	Ⅶ	Ⅷ	Ⅶ	Ⅶ	Ⅵ	Ⅶ	Ⅴ	Ⅶ	Ⅳ	Ⅴ	Ⅲ	Ⅳ
HRA	Ⅶ	Ⅷ	Ⅵ	Ⅶ	Ⅵ	Ⅶ	Ⅳ	Ⅶ	Ⅳ	Ⅴ	Ⅲ	Ⅳ

According to Table 10, both SK-70 and SBS-modified asphalt binders showed a significant decrease in adhesion after aging. The addition of recycling agent improved asphalt adhesion to a certain extent, but the adhesion between SBS-modified asphalt and aggregate was better than that of ordinary SK-70 asphalt. With the increase of BRA content, the adhesion of recycled asphalt presented a trend of slowly increasing first and then gradually decreasing. This was mainly because when the BRA content was too high, the flowability of recycled asphalt remarkably increased and its viscosity was on the contrary, and it showed a “too soft” phenomenon, thus resulting in a decline in its adhesion. Further, it can be found that among the three aggregates, limestone performed the best adhesion to recycled asphalt, followed by basalt, and granite had the worst adhesion to asphalt binder, which was mainly because asphalt binder was an acidic substance, while granite was acidic, resulting in poor adhesion ability according to the theory of chemical reactions.

#### 3.3.2 High temperature rheological properties analysis.

The unrecoverable creep compliance *J*_*nr*_ has a good correlation with the actual pavement rut, which can accurately and reasonably evaluate the high-temperature deformation resistance of asphalt materials [25]. The MSCR test process was as follows: loading for 1s followed by unloading for 9s at a stress level of 0.1 kPa, repeating this process for 10 cycles; subsequently, repeated creep recovery tests were conducted at a stress level of 3.2 kPa without interruption between the two stress level tests.

**Fig 7:**
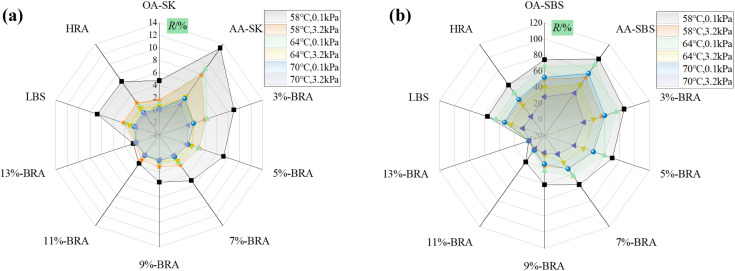
The effect of BRA dosage on the *R* value of aged asphalt. (a) Recycled aged SK-70 asphalt; (b) Recycled aged SBS modified asphalt.

**Fig 8:**
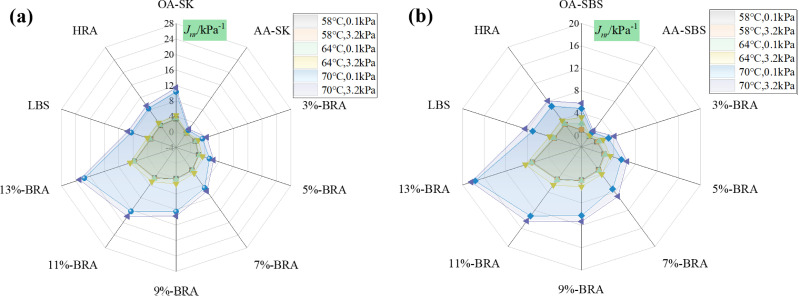
The effect of BRA dosage on the *J*_*nr*_ value of aged asphalt. (a) Recycled aged SK-70 asphalt; (b) Recycled aged SBS modified asphalt.


To evaluate the effect of BRA on the high-temperature rheological properties of aged asphalt, MSCR tests were conducted on asphalt samples after short-term aging at two stress levels of 0.1 kPa and 3.2 kPa. The creep recovery rate *R* and unrecoverable creep compliance *J*_*nr*_ were used to evaluate the high-temperature rheological properties of asphalt samples, and the calculation formulas for *R* and *J*_*nr*_ are shown in (1) and (2). The specific test results are shown in Figs 7-8.

$$R=\frac{\varepsilon_{P}-\varepsilon_{u}}{\varepsilon_{p}}\times \text{1}\text{0}\text{0}\%$$ (1)

$$J_{nr}=\frac{\varepsilon_{u}}{\sigma }$$ (2)

Where: *ε*_*p*_ — peak strain,%; *ε*_*u*_ — unrecovered strain,%; *σ* — Test stress, kPa.

Figs 7-8 show the test results of *R* and *J*_*nr*_ values for different asphalt samples at different temperatures and stress levels. The *R* represents the creep recovery ability of asphalt binder under repeated loads, and a larger *R* indicates that the asphalt binder has a better creep recovery ability, thus the rutting occurs less likely. The rutting phenomenon is caused by the accumulation of unrecoverable strain, and the smaller the *J*_*nr*_, the better the high-temperature rutting resistance of asphalt [26]. As shown in Fig 7, after aging, the *R* value of asphalt binder increased significantly, while the addition of BRA can sensibly reduce the *R* value. Moreover, the *R* value of asphalt binder presented a downward trend with the increasing BRA dosage, demonstrating that BRA greatly improved the creep recovery ability of aged asphalt, but its addition led to a decrease in the *R* value and rutting resistance. Therefore, the dosage of BRA should be appropriate. As shown in Fig 8, the addition of BRA significantly increased the *J*_*nr*_ value of aged asphalt, which was against the high-temperature anti-rutting performance of aged asphalt. Besides, an increase in temperature or stress level led to an increase in the *J*_*nr*_ value of recycled asphalt, which in turn reduced the high-temperature rutting resistance of the sample.

The magnitude of the relative difference in unrecoverable creep compliance (*J*_*nr-diff*_) reflects the stress sensitivity of asphalt, and its calculation formula is shown in Equation (3). *J*_*nr-diff*_ is an important indicator for determining the rheological performance stages of asphalt binder. When its value changes within 5.0%, ranges from 5.0 ~ 75.0%, and exceeds 75.0%, it corresponds to linear region, nonlinear region (without creep failure), and creep failure stage, respectively. By comprehensively considering and analyzing the *J*_*nr-diff*_ values at different temperatures, the applicable traffic grade types of asphalt binder can be further refined.


$$J_{\text{nr}-\text{diff}}=\frac{J_{nr\text{3}.\text{2}}-J_{nr\text{0}.\text{1}}}{J_{nr\text{0}.\text{1}}}$$ (3)

In the formula (3), *J*_*nr0.1*_ refers to the unrecoverable creep compliance at a stress level of 0.1 kPa, and *J*_*nr3.2*_ refers to the unrecoverable creep compliance at a stress level of 3.2 kPa.

**Fig 9:**
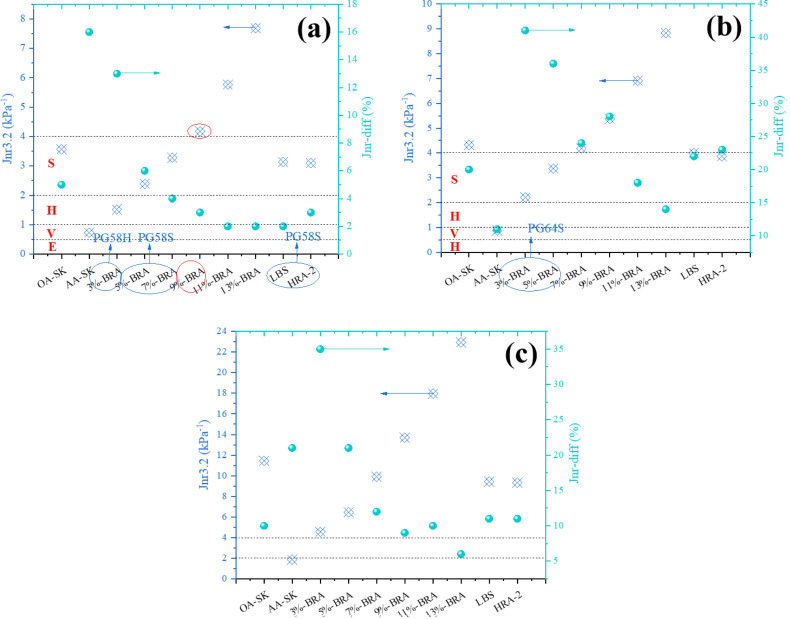
MSCR high temperature performance grading of recycled aged SK-70 asphalt. (a) 58°C; (b) 64°C; (c) 70°C.

**Fig 10:**
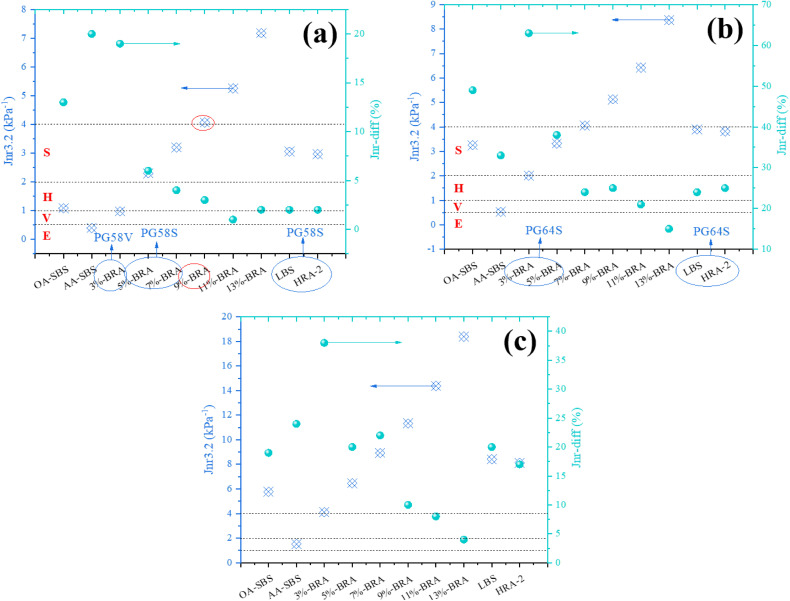
MSCR high temperature performance grading of recycled aged SBS modified asphalt. (a) 58°C; (b) 64°C; (c) 70°C.

The idea of high-temperature performance grading of modified asphalt based on MSCR tests has been proposed, which involves conducting MSCR tests on modified asphalt at actual pavement service temperatures and using the *J*_*nr3.2*_ index to proceed the performance grading [27]. According to the corresponding traffic volume level, it can be divided into standard traffic (S), heavy traffic (H), overweight traffic (V), and extremely heavy traffic (E). According to the high-temperature performance grading specified in the AASHTO M 332−23 standard, the high-temperature performance grading of different recycled asphalts is shown in Figs 9-10. Table 11 shows the regulations of AASHTO M 332−23 high-temperature performance grading for MSCR index.

**Table 11:** The regulations of AASHTO M 332−23 high-temperature performance grading for MSCR index.

PG grade			J_nr3.2_/3.2kPa^-1^	J_nr-diff_/%
58°C	64°C	70°C		
PG58S	PG64S	PG70S	≤4.5	≤75
PG58H	PG64H	PG70H	≤2.0	≤75
PG58V	PG64V	PG70V	≤1.0	≤75
PG58E	PG64E	PG70E	≤0.5	—

Following Table 11, the high-temperature performance grading of recycled asphalt was determined, and the traffic grading results are shown in Figs 9-10. As shown in the Figs 9-10, under the temperature condition of 70°C, the *J*_*nr3.2*_ values of recycled aged SK-70 asphalt and recycled aged SBS-modified asphalt both exceeded 4kPa^-1^, indicating that they were not suitable for any traffic grade and each recycled asphalt have exceeded the nonlinear viscoelastic range. At 64°C, the high-temperature grading of 3%-BRA and 5%-BRA recycled aged SK-70 asphalt, as well as recycled aged SBS-modified asphalt, was all PG64S, while the original SK-70 asphalt was not suitable for any traffic grade, and the high-temperature grading of the original SBS-modified asphalt was PG64S. Under the condition of 58°C, the high-temperature grading of 3%-BRA recycled aged SK-70 asphalt was PG58H, the high-temperature grading of 3% BRA recycled aged SBS-modified asphalt was PG58H, the high-temperature grading of 5%-BRA and 7%-BRA recycled aged SK-70 asphalt and recycled aged SBS-modified asphalt was PG58S, while the high-temperature grading of the original SK-70 asphalt and SBS-modified asphalt were PG58S and PG58H, respectively. Comprehensive analysis indicated that the recycled asphalt with suitable BRA dosage was suitable for a wider range of traffic grades than the original asphalt. Therefore, from the perspective of ensuring the high-temperature stability of the road surface, both types of aged asphalt binder achieved good recycling effects. Further, Figs 9-10 showed that the *J*_*nr-diff*_ of all the recycled asphalt binders was less than 75%, meaning that the recycled asphalt binders exhibited good creep recovery ability and did not reached the creep failure stage under increased-load condition. With the increase of BRA dosage, the J_nr-diff_ gradually recued as a whole, for example, as for aged SK-70 asphalt binder, the addition of 5%, 7%, 9%, 11% and 13% BRA resulted in a decrease of 53.8%, 33.3%, 25.%, 33.3% and 0% in J_nr-diff_ at 58°C, indicating that the incremental BRA dosage lowered the sensitivity of aged asphalt binder to vehicle load stress, which reflected that the growing BRA dosage gradually restored the viscoelastic properties of the aged asphalt binder.

#### 3.3.3 Fatigue resistance performance analysis.

**Fig 11:**
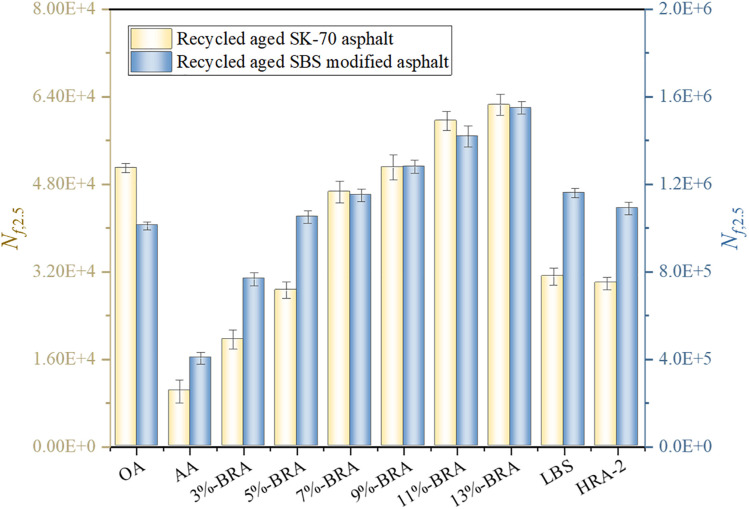
Effect of BRA on fatigue performance of aged asphalt.

Road fatigue damage is the phenomenon of the generation, propagation, and connectivity of micro-cracks within a certain volume range, with high localization and nonlinear characteristics. The fatigue performance of asphalt binder and its interfacial adhesion to the aggregate are the main factors affecting the fatigue life of asphalt pavement. Therefore, the fatigue behaviors of asphalt binders are of great significance for the long-term durability of asphalt pavement. Due to the fact that fatigue damage generally occurs at the intermediate layer position between 10°C and 30°C [28,29], the LAS test was conducted on long-term aged samples at 25°C by using DSR in this section. The fatigue life parameter *N*_*f,2.5*_ of the sample was obtained by fitting and calculating the test results using Excel software, so as to analyze the effect of BRA on the fatigue resistance of aged asphalt. Here, *N*_*f,2.5*_ refers to the fatigue life of asphalt binder at the expected strain of 2.5%. The specific test results are shown in Fig 11.

As shown in Fig 11, the fatigue life of asphalt remarkably decreased after aging, which can lead to the fatigue cracking of asphalt pavement. The addition of BRA prominently improved the fatigue life of aged asphalt, and with the increasing BRA dosage, the anti-fatigue performance of aged asphalt gradually enhanced, indicating that the addition of BRA greatly upgraded the anti-fatigue damage performance of aged asphalt and achieved good recycling effect. However, overly high BRA content may lead to fatigue performance decline. This was chiefly because BRA was a small polar and low-viscosity molecule that can supplement the lightweight components lost in asphalt binder due aging and restore the fatigue performance of aged asphalt, while a large amount of BRA will cause excessive softening of asphalt binder, thereby reducing its fatigue performance. Besides, when the BRA dosage was 7% −9%, the fatigue life of aged SK-70 asphalt could be restored to the same level as the original SK-70 asphalt; When the BRA dosage was 3% −5%, the fatigue resistance of aged SBS-modified asphalt can be restored to the same level as the original SBS-modified asphalt. Meanwhile, compared to LBS and HRA, BRA performed better in improving the fatigue resistance of aged asphalt.

As mentioned above, the fatigue performance of recycled asphalt binder was intimately associated with the BRA dosage and rejuvenator type. In order to further understand the influence of BRA dosage and rejuvenator type on the fatigue performance of recycled asphalt, a multivariate analysis of variance (ANOVA) was conducted with a confidence level of 95%, and the results are shown in Table 12.

**Table 12:** ANOVA results on the effects of BRA dosage and rejuvenator type on the fatigue performance of recycled asphalt.

Asphalt types	Error sources	Sum of squares	Freedom	Mean square error	*F*	Sig. *p*
SK-70#	Intercept	4.777E + 9	1	4.777E + 9	1814.692	0.000
	Rejuvenator dosage	4.893E + 9	5	9.786E + 8	371.768	0.000
	Rejuvenator type	8.245E + 8	2	4.122E + 8	156.608	0.000
	Error	4.212E + 7	16	2.632E + 6	–	–
SBS	Intercept	5.645E + 12	1	5.645E + 12	7457.921	0.000
	Rejuvenator dosage	1.170E + 12	5	2.340E + 11	309.146	0.000
	Rejuvenator type	8.109E + 10	2	4.055E + 10	53.568	0.000
	Error	1.211E + 10	16	7.569E + 8	–	–

Following the theory of ANOVA, a *p*-value less than or equal to 0.05 means that this factor has a significant impact on the dependent variable, and the larger the *F*-value, the more remarkable its impact on the dependent variable [22]. Thus, it was found from Table 12 that both dosage and type of rejuvenator notably affected the fatigue performance of neat and SBS-modified recycled asphalt binders, and rejuvenator dosage showed a larger influence on their fatigue performance, compared to rejuvenator type.

#### 3.3.4 Aging resistance analysis.

**Fig 12:**
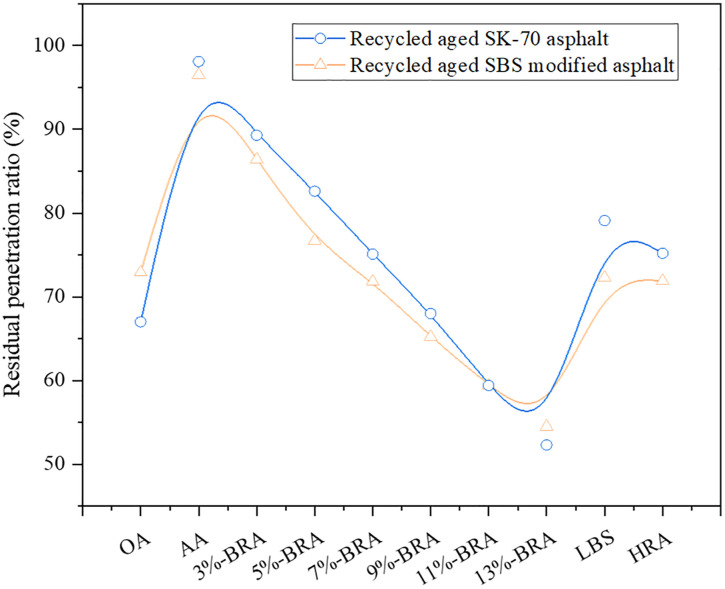
Analysis on aging resistance of recycled aged SK-70 and SBS-modified asphalts.

In order to evaluate the influence of BRA on the anti-aging performance of aged asphalt, the different amounts of BRA, LBS and HRA were used to rejuvenate different types of aged asphalt binders to prepare different recycled asphalt samples. Next, the film oven aging test (TFOT) was carried out on the recycled asphalt samples, and the residual penetration ratio (the ratio of penetration after aging to that before aging) of the samples were tested to evaluate the anti-aging performance of recycled asphalt. The greater residual penetration ratio indicates the more favorable anti-aging performance of asphalt. The specific test results are shown in Fig 12.

It can be seen from Fig 12 that after aging, the residual penetration ratio of asphalt significantly increased, indicating that the aging resistance of aged asphalt was much higher than that of the original asphalt. This was chiefly because the aging process of aged asphalt was deeper after 6h of aging in the rotary film oven, at which aging time, the volatile light components in the asphalt have also been volatilized, so the impact of re-aging on the sample was small, leading to fewer changes in aging indicators. Secondly, with the increase of the amount of BRA, the residual penetration ratio descended, signifying that the addition of BRA resulted in the decline of the anti-aging performance of the sample. However, when the BRA dosage was 7% −9%, the residual penetration ratio was higher than the level of the original asphalt, which demonstrated that although the addition of BRA reduced the aging resistance of the sample, the anti-aging performance of the bio-recycled aged SK-70 asphalt with the content of 7% −9% was still better than the original asphalt. Meanwhile, when the BRA dosage was 3% −5%, the residual penetration ratio was higher than that of the original SBS-modified asphalt, illustrating that when the BRA dosage was 3% −5%, the anti-aging performance of recycled aged SBS-modified asphalt was better than that of the original SBS-modified asphalt. In addition, whether for aged SK-70 asphalt or aged SBS-modified asphalt, the aging resistance of bio-recycled asphalt with the same dosage was better than LBS recycled asphalt and HRA recycled asphalt.

#### 3.3.5 Determination of optimal bio-recycling agent dosage.

For determining the optimal bio-recycling agent dosage, based on the recycling efficiency analysis, the above evaluation indexes, which included non-aged, aged, BRA-added, LBS-added, HRA-added neat and SBS-modified asphalt binder. The extracted results are shown in Table 13.

**Table 13:** Extracted results of evaluation indexes.

Items	Adhesion (Limestone)		High-temperature				Anti-fatigue		Aging resistance	
	SK (6 min)	SBS (6 min)	SK (58°C, 0.1kPa)		SBS (58°C, 0.1kPa)		SK	SBS	SK	SBS
	Level	Level	*R*	*J*_*nr*_	*R*	*J*_*nr*_	*N*_*f*,2.5_	*N*_*f*,2.5_	Residual penetration ratio	Residual penetration ratio
OA	Ⅸ	Ⅹ	4.69	3.401	74.12	0.957	51000	1010000	67.0	72.9
AA	Ⅴ	Ⅶ	13.21	0.632	97.33	0.332	10100	405000	98.1	96.5
3%-BRA	Ⅶ	Ⅷ	9.01	1.346	86.21	0.821	19610	766000	89.3	86.4
5%-BRA	Ⅶ	Ⅷ	7.21	2.258	70.56	2.177	28600	1050000	82.6	76.7
7%-BRA	Ⅷ	Ⅸ	5.08	3.157	55.36	3.068	46600	1150000	75.1	71.8
9%-BRA	Ⅷ	Ⅸ	3.58	4.068	41.21	3.963	51100	1280000	68.0	65.2
11%-BRA	Ⅷ	Ⅷ	1.68	5.621	20.56	5.189	59600	1420000	59.4	59.4
13%-BRA	Ⅶ	Ⅶ	0.53	7.532	1.15	7.021	62500	1550000	52.3	54.5
LBS	Ⅶ	Ⅷ	6.8	3.069	56.11	2.986	31100	1160000	79.1	72.3
HRA	Ⅶ	Ⅷ	6.57	3.001	57.26	2.898	29900	1090000	75.2	71.9

According to Table 13, when the BR content was 7% −9%, the recycled sample reached the highest adhesion level, but at this time, its adhesion level was still slightly lower than that of the original asphalt. Compared to LBS and HRA, at the same recycling agent dosage (9%), the adhesion level of BRA recycled samples was generally higher. It can also be seen that when the BRA dosage was 7% −9% and 3% −5%, respectively, the *R*, *J*_*nr*_ and *N*_*f*,2.5_ values of aged SK-70 asphalt and aged SBS modified asphalt could be basically restored to the same level as the original asphalt, and compared to LBS and HRA, at the same dosage, BRA had better recycling effects on the high-temperature rutting resistance and anti-fatigue of aged asphalt, but the dosage should be appropriate. Further, when the BRA dosage was 7% −9% and 3% − 5%, respectively, the residual penetration ratio of aged SK-70 and SBS-modified asphalt binders was higher than the level of the original asphalt, illustrating that the anti-aging performance of bio-recycled aged SK-70 asphalt with the content of 7% −9% and bio-recycled aged SBS-modified asphalt with the content of 3% −5% was better than that of the original asphalt.

To sum up, by comprehensively analyzing the influence of BRA on the basic physical properties, high and low temperature rheological properties, fatigue resistance and aging resistance of aged asphalt, the optimal BRA dosage for different aged asphalts can be determined — for aged SK-70 asphalt, it is recommended that the optimal BRA dosage should be 7% −9%; for aged SBS modified asphalt, it is recommended that the optimal BRA dosage should be 3% −5%.

### 3.4 Microscopic mechanism analysis of bio-recycled asphalt

#### 3.4.1 Temperature sensitivity analysis.

**Fig 13:**
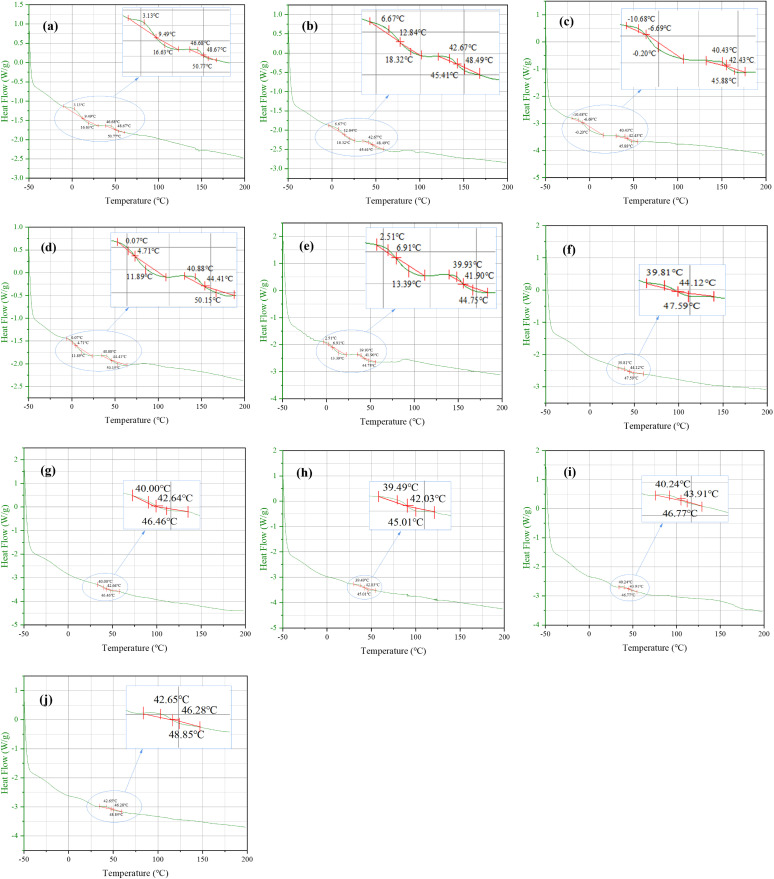
DSC test results of recycled asphalt samples. (a) OA-SK; (b) AA-SK; (c) BRA-SK; (d) LBS-SK(e) HRA-SK; (f) OA-SBS; (g) AA-SBS; (h) BRA-SBS; (i) LBS-SBS; (j) HRA-SBS.

In the actual practice of asphalt pavement recycling engineering, during the mixing, paving and application stages of asphalt mixtures, the asphalt binder undergoes temperature changes from −50°C to 200°C. If the thermal stability of the binder is poor, the temperature variations will have a serious impact on its service performance. In view of this, the temperature susceptibility of recycled asphalt sample was evaluated through differential scanning calorimetry (DSC). By conducting DSC experiments to obtain the DSC curve of the sample, two important parameters could be obtained from the DSC curve: (1) the temperature range of the endothermic peak — the temperature range in which the aggregation state undergoes transformation, the larger the range, the larger the temperature range in which the thermal properties of the material are unstable, indicating poorer thermal stability; and (2) the area of the endothermic peak — the heat absorbed by the transformation of the aggregated state, the larger the area, the larger the components that undergo changes within this temperature range, indicating the more unstable properties of material and poorer thermal stability. In order to evaluate the temperature sensitivity of bio-recycled asphalt, the DSC tests were executed on recycled asphalt samples (original SK-70 asphalt and SBS modified asphalt, aged SK-70 asphalt, aged SBS-modified asphalt, 9% BRA recycled SK-70 asphalt, 5% BRA recycled SBS-modified asphalt, 9% LBS and HRA recycled asphalt) at the optimal dosage of recycling agent. The test temperature was −50°C −200°C. The DSC test curves of the asphalt samples are shown in Fig 13, and the specific test results are shown in Table 14.

**Table 14:** Energy value of endothermic peak of recycled asphalt sample.

Asphalt type	Starting temperature (°C)	End-point temperature (°C)	Endothermic peak energy value (J/g)	Total endothermic peak energy value ΔH (J/g)
OA-SK	−7.14	9.49	0.2816	1.1195
	9.49	26.85	0.6848	
	42.51	48.67	0.0900	
	48.67	56.64	0.0631	
AA-SK	−5.42	12.84	0.3028	0.6364
	12.84	25.01	0.1847	
	39.26	48.49	0.0761	
	48.49	54.73	0.0728	
BR-SK	−17.21	−6.69	0.2544	0.8253
	−6.69	12.15	0.3993	
	37.16	42.43	0.0614	
	42.43	52.44	0.1102	
LBS-SK	−5.04	4.71	0.1143	0.7764
	4.71	21.50	0.4280	
	36.20	44.41	0.1258	
	44.41	57.02	0.1083	
HRA-SK	−3.13	6.91	0.1375	0.6639
	6.91	22.84	0.3336	
	35.25	41.90	0.0683	
	41.90	52.63	0.1245	
OA-SBS	34.87	44.12	0.0691	0.1444
	44.12	53.20	0.0753	
AA-SBS	32.19	42.64	0.0457	0.1193
	42.64	52.44	0.0736	
BR-SBS	32.77	42.03	0.0787	0.1136
	42.03	51.10	0.0349	
LBS-SBS	34.30	43.91	0.0727	0.1043
	43.91	51.67	0.0316	
HRA-SBS	34.30	46.28	0.1502	0.2056
	46.28	54.54	0.0554	

Table 14 shows the range of endothermic peak and the changes in energy values of asphalt samples before and after aging and recycling. According to the Table, whether it was SK-70 asphalt or SBS-modified asphalt, the total endothermic peak energy value ΔH after aging decreased, indicating that the thermal stability of aged asphalt was better than that of the original asphalt. This was mainly due to the fact that during the aging process of asphalt, the volatile light components reduced and the heavy components increased, causing the asphalt to become harder and more elastic, resulting in better thermal stability. In addition, the ΔH of the three types of recycled asphalt samples were lower than those of the original asphalt, for example, the ΔH of BR-SK sample was 26.28% lower than that of BR-SK sample and the ΔH of BR-SBS sample was 21.33% less than that of OA-SBS sample, demonstrating that recycled asphalt was less sensitive to temperature and possessed better thermal stability. The ΔH values of SBS modified asphalt samples was generally lower than that of SK-70 asphalt, illustrating that SBS modified asphalt had more favorable thermal stability. For aged SK-70 asphalt, the ΔH values of the recycled samples BRA-SK, LBS-SK and HRA-SK were 0.8253, 0.7764 and 0.6639 J/g, respectively, that is, the ΔH values were ranked in descending order: BRA-SK > LBS-SK > HRA-SK. This signified that the thermal stability of the recycled samples with HRA recycling was stronger than that of BRA and LBS recycled asphalt, which was chiefly attributable to the higher content of light components (aromatic components) in BRA and LBS, leading to slightly poorer thermal stability but still meeting the requirements. For aged SBS modified asphalt, the HRA-SBS sample exhibited the highest ΔH value (reached 0.2056 J/g), followed by BRA-SBS and LBS-SBS, implying that the thermal stability of the recycled samples with HRA recycling was poor. This was mainly due to the favorable thermal stability of SBS modified asphalt itself, and its aging mechanism differed from that of the matrix asphalt, so the degradation of SBS modifier needed to be considered. Therefore, there were certain differences in the performance of different aged asphalts after adding recycling agent, resulting in the phenomenon that HRA-SK asphalt possessed better thermal stability while HRA recycled SBS-modified asphalt performed poorly in thermal stability.

Further, for multiphase polymer materials, the ΔH values can reflect the homogeneity of the polymer materials and the compatibility between polymer and host materials — a smaller peak energy value commonly meant that the polymer-modified material was more homogeneous and had better compatibility with the host material. It was found from Table 11 that as for SBS-modified asphalts, the peak energy values of OA-SBS, AA-SBS and BR-SBS were ordered by OA-SBS > AA-SBS > BR-SBS, which indicated that the aging compelled SBS-modified asphalt more uniform and improved the compatibility between SBS modifier and asphalt binder. This was mainly attributable to the growing solubility caused by the degradation of SBS modifier under aging; the addition of BRA further reduced peak energy, reflecting that the rejuvenation of BRA upgraded the homogeneity of aged SBS-modified asphalt and the compatibility of SBS modifier.

Besides, considering the temperature range where the endothermic peak occurred, for SK-70 asphalt aged and recycled samples, there were two endothermic peak ranges, around 0°C and around 50°C, respectively; For SBS modified asphalt samples, there was only one endothermic peak interval that distributed around 50°C. This indicated that compared to SK-70 asphalt samples, the temperature range for phase transition of SBS modified asphalt samples was smaller, demonstrating that the temperature range for material instability was smaller, indirectly verifying the better thermal stability of SBS modified asphalt samples before and after aging and recycling. In summary, the samples recycled by the three types of recycling agents all exhibited good thermal stability, and the thermal stability of the recycled samples was basically the same as or higher than that of the original asphalt. Additionally, it was worth noting that the analysis results just described were obtained based on the testing conditions of the DSC experiment. Considering the time dependency of DSC test as well as its commonly in use process to measure thermal stability and temperature sensitivity, the utilizing new approaches in uniform normalizing the recycled asphalt binders in large scale industrial application could be a future trend. For example, as for the DSC test, normalizing the large-scale data can be conducted according to the four steps below: (1) the DSC test parameters (temperature rate, ΔH) of different recycled asphalt binders were obtained at various sampling durations; (2) the most appropriate combination of DSC parameters was screened through a dynamic multi-gated band filtering procedure based on the mode and long-term average statistics; (3) the most appropriate parameter combination was normalized via using statistical normalizing (such as linear, nonlinear and logarithmic methods); (4) a unified database was established by the PostreSQL platform [30].

#### 3.4.2 Microscopic phase morphology.

**Fig 14:**
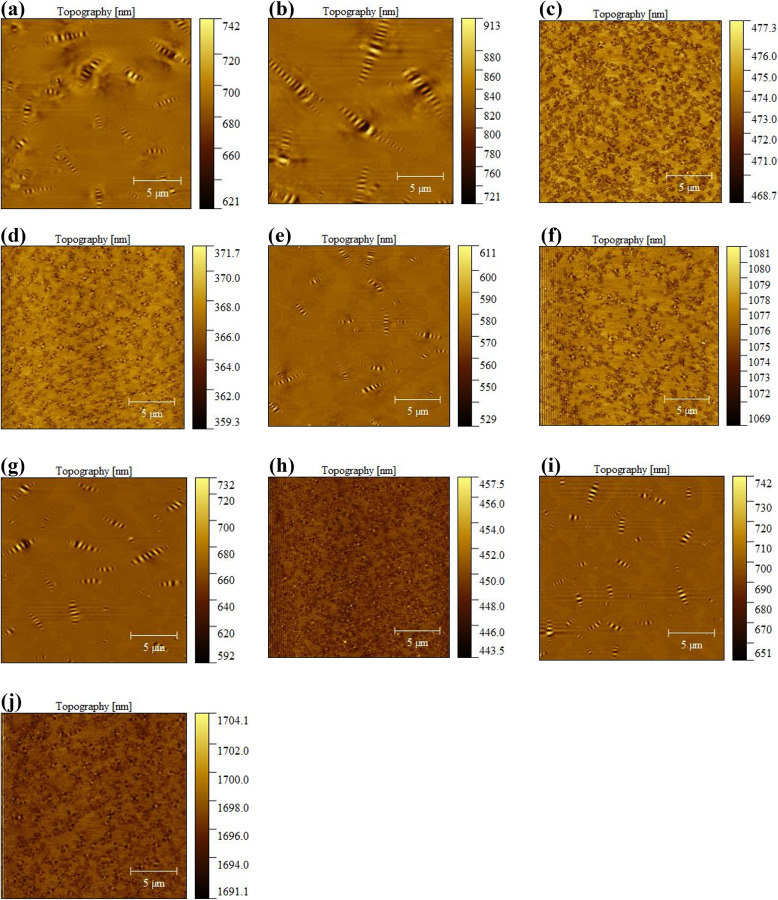
Microscopic morphology structure of asphalt samples before and after aging and recycling. (a) OA-SK; (b) AA-SK; (c) OA-SBS; (d) AA-SBS; (e) BRA-SK; (f) BRA-SBS; (g) LBS-SK; (h) LBS-SBS; (i) HRA-SK.; (j) HRA-SBS.

The microstructure of asphalt is closely related to its macroscopic properties. In order to explore the changes in microstructure of asphalt before and after aging and recycling, and analyze the recycling effect of BRA on the microstructure characteristics of aged asphalt, the microstructure characteristics of ten samples were measured through atomic force microscopy (AFM) in this section, with the test results shown in Fig 14.

**Fig 15:**
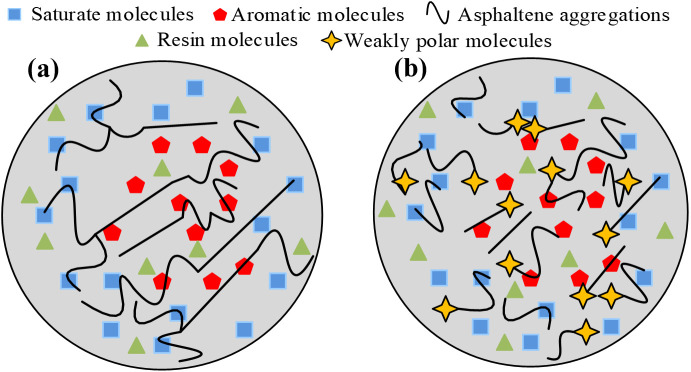
Schematic diagram of BRA depolymerizing asphaltene. (a) Before adding BRA; (b) After adding BRA.

From Fig 14(a)–(b), it can be seen that after aging, the number of “bee-structures” in SK-70 asphalt decreased but the size increased, indicating that aging promoted the development of honeycomb structures, and some honeycomb structures may even aggregate and merge under aging, leading to a decline in honeycomb number and an enlargement in size. As shown in Fig 14(e), after adding BRA, the number and size of honeycomb structures in aged asphalt reduced, mainly due to the dissolution effect of BRA on asphaltene closely related to honeycomb structures in asphalt — as shown in Fig 15, after asphalt aging, its asphaltene aggregated, and after adding BRA, BRA was a weakly polar molecule substance that can depolymerize the asphaltene, thereby causing the microstructure of asphalt binder to undergo the just-mentioned variations. Meanwhile, this also illustrated that BRA possessed a certain dispersing effect on the aggregation of gel particles in asphalt, thereby restoring the colloidal structure of asphalt.

**Fig 16:**
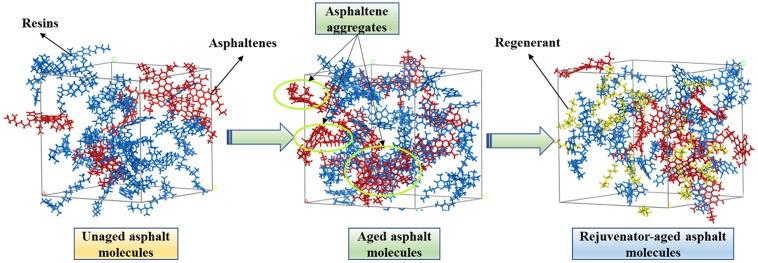
Molecular mechanism schematic of recycling aged asphalt.

As shown in Fig 14(g)–(i), the addition of LBS and HRA to aged SK-70 asphalt resulted in a growth in the number of honeycomb structures but a decline in honeycomb size, demonstrating that these two recycling agents possessed a favorable recovery effect on the colloidal structure of aged asphalt. Comparing Fig 14(e), 14(g) and 14(i), it can be found that there was a circle of enveloping phase around the honeycomb structure in the BRA-SK sample. This was because compared to LBS and HRA, BRA not only regulated the aromatic content in aged asphalt, but also performed a certain dissolving effect on the aggregated asphaltene, causing it to re-disperse in the asphalt colloid dispersion system and restore the colloid structure of asphalt. From Fig 14(c)–(d), it was found that compared to SK-70 asphalt, the honeycomb structure of SBS modified asphalt was not obvious. Research showed that strongly polar asphaltene or microcrystalline wax was closely related to the formation of honeycomb structure in asphalt micro-morphology, and some asphaltene or asphalt with low microcrystalline wax content hardly contained honeycomb structure. So, this may be due to the low content of asphaltene or microcrystalline wax in the matrix asphalt used in the preparation of SBS modified asphalt, for example, there is no honeycomb structures in the micro-morphology of Karamay matrix asphalt [31]. In addition, the microstructure of SBS modified asphalt hardly displayed significant variations after aging, indicating that the microstructure of SBS modified asphalt was not remarkably affected by aging. According to Fig 14(f)-(j), it can be seen that after adding the recycling agent, there was little change in the microstructure of SBS modified asphalt before and after aging, illustrating that all three recycling agents demonstrated good recycling effects on the microstructure of aged asphalt. In summary, at the microscopic perspective, the rejuvenation of aged asphalt was the process of restoring the microstructure of aged asphalt, and during this process, the depolymerization of asphaltene aggregates and the restoration of microstructure were the typical recycling behaviors under the intervention of rejuvenator, with the molecular mechanism shown in Fig 16.

**Fig 17:**
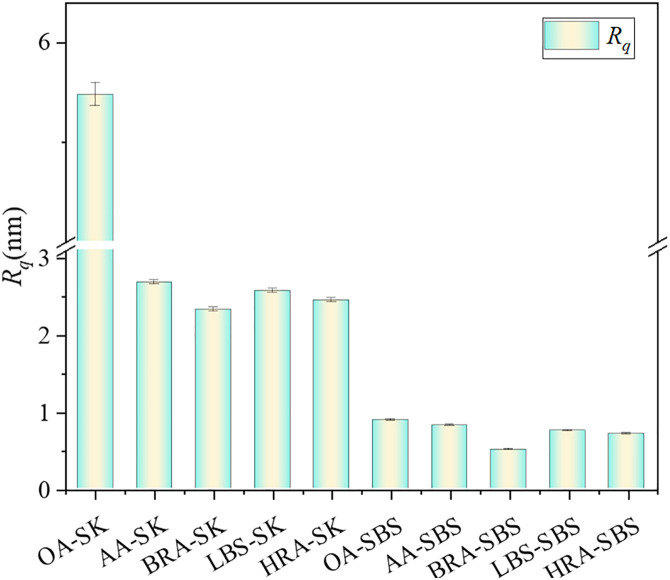
Changes in roughness of asphalt before and after aging and recycling.


To quantitatively analyze the microstructure changes of different asphalt before and after aging and recycling, and evaluate the recycling effect of BRA on its morphology and structure, the Gwyddion software was used to calculate the root mean square roughness (*R*_*q*_), and the specific calculation formula is shown in Equations (4)–(5). The calculation results are shown in Fig 17.

$$R_{q}=\sqrt{\frac{\iint {\left[h(x,y)-h_{\text{0}}\right]}^{\text{2}}ds}{\iint ds}}$$ (4)

$$h_{\text{0}}=\frac{\iint h(x,y)ds}{\iint ds}$$ (5)

Research has shown that the lower asphaltene content promotes the formation of honeycomb structures in the microstructure of asphalt, while the higher asphaltene content inhibits the formation of honeycomb structures [32]. It can be seen from Fig 17 that the *R*_*q*_ of SK-70 asphalt decreased after aging, which was mainly due to the poor anti-aging performance of SK-70 asphalt, significantly increasing the asphaltene content after aging, so it inhibited the formation of honeycomb structure in asphalt, resulting in flat and smooth asphalt micro-surface and reduced roughness. The addition of recycling agents also led to a slight decline in the surface roughness of asphalt, which was chiefly attributable to the dissolution effect of recycling agents on the honeycomb structure of asphalt; meanwhile, the BRA performed a stronger solubility for honeycomb structure in aged asphalt than LBS and HRA. Therefore, the *R*_*q*_ of BRA-SK samples was lower and the surface was flatter and smoother. In addition, although there was no honeycomb structure in the microstructure of SBS modified asphalt, from the perspective of surface roughness, the *R*_*q*_ changes of SBS modified asphalt before and after aging and recycling were consistent with those of SK-70 asphalt.

#### 3.4.3 Microscopic adhesion force.

**Fig 18:**
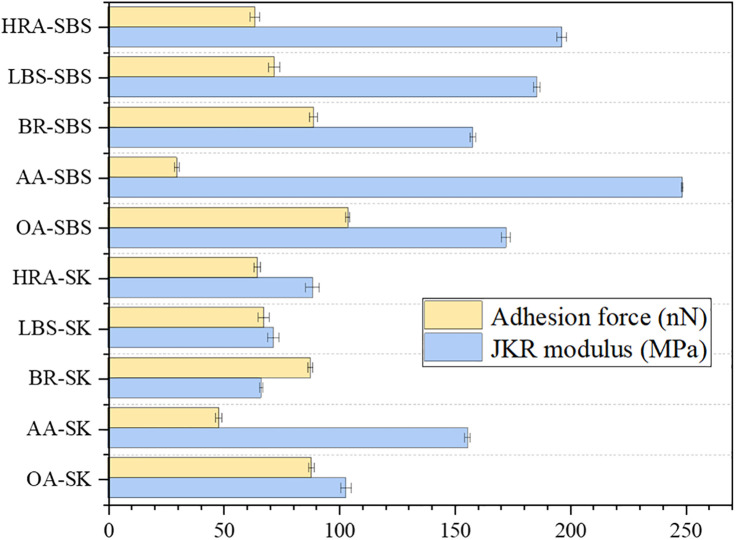
Micro-adhesion test results of different recycled asphalt samples.

Section 3.3.1 used the boiling method to investigate the adhesion variation laws of aged asphalt with the addition of BRA. It was found that after aging, the adhesion of asphalt significantly decreased, leading to a decline in the road performance of mixture, especially the water stability; and the addition of recycling agents, especially BRA, remarkably improved the adhesion of recycled asphalt. Given that the water boiling method is a macroscopic experimental method, so the AFM technology was employed to test the force-displacement curves of different recycled asphalt samples and aggregates in this section, and the JKR mechanical model was employed for fitting to calculate the JKR modulus and adhesion force of asphalt samples, so as to explore the recycling effect of BRA on the adhesion of aged asphalt at the microscopic scale. The specific results are shown in Fig 18.

**Fig 19:**
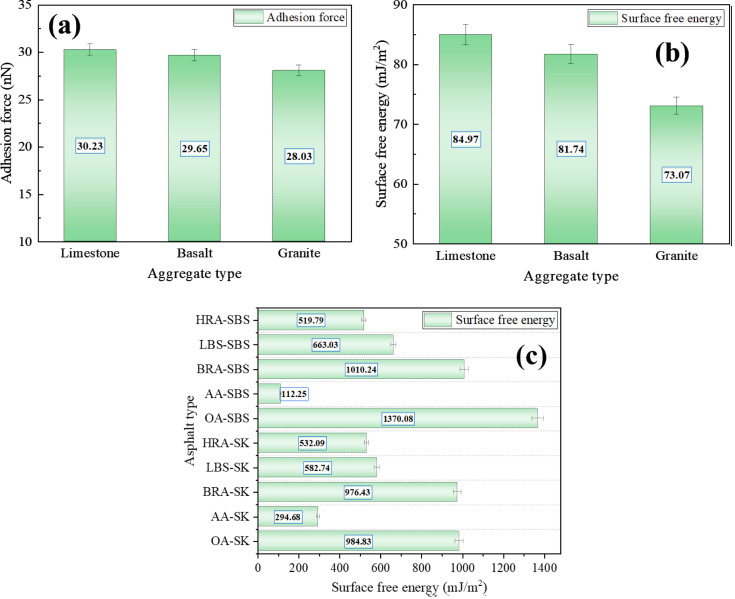
Microscopic adhesion force and surface free energy. (a) Adhesion force of aggregate; (b) Surface free energy of aggregate; (c) Surface free energy of asphalt.

**Fig 20:**
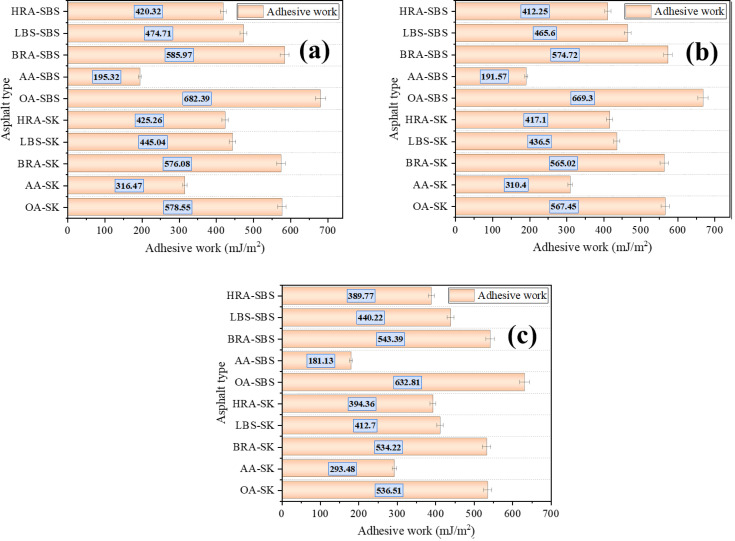
Microscopic adhesive work of asphalt-aggregate interface. (a) Asphalt-limestone aggregate; (b) Asphalt-basalt aggregate; (c) Asphalt-granite aggregate.


However, the micro-adhesion force test results shown in Fig 18 refer to the adhesion force between asphalt and AFM test probes, rather than the adhesion force between asphalt and aggregates. Therefore, in order to scientifically and reasonably analyze the recycling effect of BRA on the adhesion of aged asphalt (asphalt-aggregate interface), the AFM technology was used to measure the micro adhesion force of limestone, basalt, and granite aggregates, and calculate the surface free energy of asphalt and aggregate. The test calculation results are shown in Fig 19. Based on this, the interfacial adhesive work between asphalt and aggregate was calculated according to Formula (6), and the specific calculation results are shown in Fig 20. *γ*_*a*_ and *γ*_*s*_ (mJ/m^2^) respectively refer to the surface free energy of asphalt and aggregate, while *W*_*as*_ refers to the adhesiv ework at the asphalt-aggregate interface.

$$W_{as}=\text{2}\sqrt{\gamma_{a}\gamma_{s}}$$ (6)

Fig 19 showed that the micro-adhesion force and surface free energy of limestone were the highest, while those of granite aggregate were the lowest, indicating that limestone had excellent adhesion capacity. According to Fig 20, the interfacial *W*_*as*_ between asphalt and different aggregates manifested a consistent trend with the asphalt types, and after aging, the *W*_*as*_ of asphalt significantly decreased, demonstrating that aging prominently reduced the adhesion of asphalt materials. After adding the recycling agent, the *W*_*as*_ at the asphalt-aggregate interface markedly improved, signifying that the recycling agent enhanced the adhesion between aged asphalt and aggregate, and represented a notable recycling effect. Compared to the two commercially available recycling agents, BRA behaved a more remarkable effect on improving adhesive work, and it could restore the *W*_*as*_ of the aged asphalt-aggregate interface to the level of the original asphalt, implying that BRA possessed a better recycling and recovery effect on the adhesion of aged asphalt. In addition, it can be seen from the Fig 20 that BRA possessed a satisfactory recycling efficiency on the micro-adhesion of aged SK-70 asphalt and aged SBS modified asphalt, which was consistent with the results of macro-adhesion tests.

**Fig 21:**
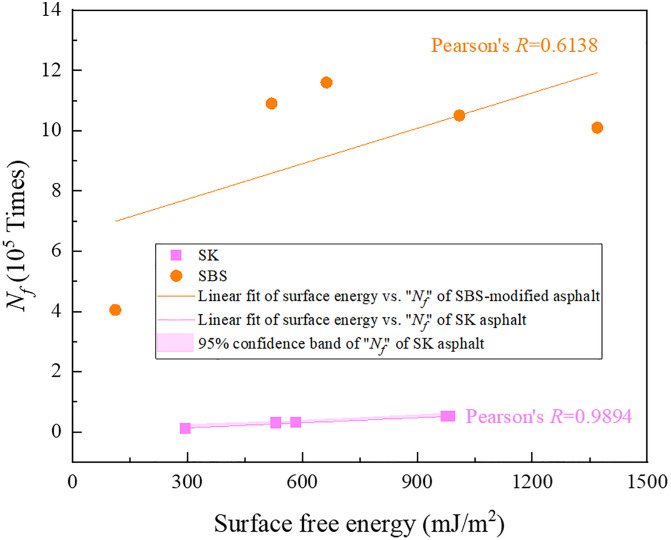
Correlation analysis results between surface free energy of asphalt and fatigue life.

Besides, the micro behaviors of a material determine its macro properties, and the correlation analysis between surface free energy of asphalt and its fatigue life was conducted on relevant data for further analysis, with the results shown in Fig 21. It can be seen that as for neat recycled asphalt binder, there existed favorable linear Pearson correlation between surface free energy and fatigue life *N*_*f*_ (the Pearson correlation coefficient *R* reached 0.9894), which reflected that the recovery of thermodynamic properties of aged neat asphalt binder after adding the rejuvenator was an important contributing factor to improving its fatigue life. However, no good linear correlation was found between surface free energy of SBS-modified recycled asphalt binder and its fatigue life, and further exploration was needed by increasing the number of samples in the later stage. Besides, the presented regression in this study mathematically can be enhanced by upcoming updates, and in future studies, there is potential room to strengthen the capability of the achieved correlations [33].

## 4 Conclusion

(1) Based on the orthogonal test method, the optimal composition ratio of BRA was determined to be base oil components: permeation components: polymer components: functional components = 120: 2: 13: 2.4. Compared with the commercial recycling agents, the BRA performed better in wettability and permeability.
(2) Compared to commercial recycling agents, at the same dosage, BRA behaved better recycling effects in restoring the adhesion, high- and low-temperature rheological properties, fatigue resistance and aging resistance of aged asphalt. According to the comprehensive recycling effects, the reasonable dosage of BRA for recycling the aged matrix asphalt was recommended to be 7%−9%, and the suggested dosage of BRA was 3%−5% for the rejuvenation of aged SBS-modified asphalt binder.
(3) Recycled asphalt binders had differentiated thermal stability and the thermal stability level of recycled samples was basically the same as or higher than that of the non-aged asphalt.
(4) The rejuvenation of aged asphalt binder decreased the number and size of bee-shaped structures in microscopic phase morphology, and meanwhile slightly descended its surface roughness. Compared to commercial recycling agents, BRA not only regulated the aromatic content in aged asphalt, but also had a certain dissolution effect on the aggregated asphaltene, causing it to be redispersed in the asphalt colloid dispersion system, thereby restoring the colloid structure of asphalt and achieving the recycling of aged asphalt.
(5) Rejuvenators possessed noticeable recovery capability on the micro-adhesion of aged asphalt, and compared to two commercial recycling agents, the BRA demonstrated a better recovery effect on the micro-adhesion of aged asphalt.

As mentioned above, this research successfully investigated the composition ratio, rejuvenation efficiency and micro recycling mechanism of BRA, which provided solid theoretical support for the promotion and application of BRA. Nevertheless, the field-scale application effects of BRA and the long-term durability of BRA recycled asphalt pavement have not been yet assessed, which will be the next research topic.
